# Green synchronous spectrofluorimetric method for the simultaneous determination of agomelatine and venlafaxine in human plasma at part per billion levels

**DOI:** 10.1038/s41598-022-26827-2

**Published:** 2022-12-29

**Authors:** Galal Magdy, Fathalla Belal, Asmaa Kamal El-Deen

**Affiliations:** 1 Pharmaceutical Analytical Chemistry Department, Faculty of Pharmacy, Kafrelsheikh University, Kafrelsheikh, 33511 Egypt; 2 Pharmaceutical Analytical Chemistry Department, Faculty of Pharmacy, Mansoura University, Mansoura, 35516 Egypt

**Keywords:** Chemistry, Analytical chemistry, Fluorescent probes

## Abstract

A novel sustainable, simple, sensitive, and green spectrofluorimetric method was developed for the concurrent estimation of venlafaxine and agomelatine in pharmaceuticals and biological fluids. The method relies on synchronous fluorescence spectroscopy, where venlafaxine and agomelatine were measured at 276 and 328 nm, respectively, using Δλ of 20 nm. The potential factors affecting the fluorescence intensity were optimized by the one-factor-at-a-time (OFAT) strategy, where synchronous fluorescence intensity was significantly enhanced using a 1% *w/v* sodium dodecyl sulfate micellar system. The method was fully validated and exhibited excellent linearity (*r*^2^ > 0.999 for both drugs) with very low limits of detection (LODs) in the range of 0.14–0.84 ng/mL. Consequently, the proposed approach was efficiently adopted to analyze the co-administered drugs in their pharmaceuticals and in spiked human plasma with excellent % recovery between 97.4 and 102.2%. Finally, the method's greenness was evaluated using different metric tools, including Green Analytical Procedure Index (GAPI) and Analytical GREEnness (AGREE), which proved its excellent greenness.

## Introduction

Depression (a common mental disorder) is one of the most prevalent main psychiatric disorders. Major depressive disorders are more common in those who have suffered a stroke (10–27%), myocardial infarction (40–65%), and cancer (20–25%). Antidepressant drugs have been used to treat all types of severe depressive disorders^1^. Over the past few years, antidepressant prescriptions have significantly increased in Egypt. The selective serotonin-norepinephrine reuptake inhibitors (SNRIs) and the serotonin-2C (5-HT2C) antagonists are two significant classes of antidepressants that are frequently prescribed in psychiatry^2^. They have clinical efficacy comparable to that of traditional tricyclic antidepressants but lack some of the negative cardiovascular and anticholinergic side effects frequently associated with these medications^3^.

**Figure 1:**
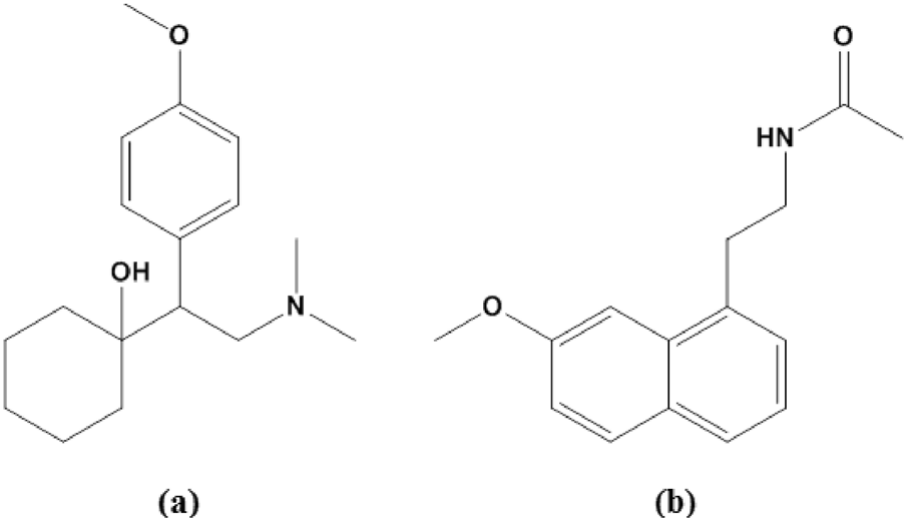
Structural formulae of (**a**) Venlafaxine, (**b**) Agomelatine.

Venlafaxine (VFX), (1-[2-(dimethylamino)-1-(4-methoxyphenyl)ethyl] cyclohexanol), is a second-generation SNRIs antidepressant drug (Fig. 1a)^4^. In comparison to the antidepressant fluoxetine, VFX has shown a rapid onset of action and improved response^5^. However, the most prevalent side effects of VFX use include depression, serotonin poisoning, seizures, or problems with heart conduction^6,7^. Agomelatine (AGM), also known as N-(2-(7-methoxy-1-naphthyl) ethyl) acetamide, is a new antidepressant medication^8^ that acts as both a 5HT-2C antagonist and an agonist on MT1/MT2 melatonergic receptors^9^ (Fig. 1b). Comparable efficacy to SSRIs like paroxetine and SNRIs like VFX has been demonstrated in clinical studies^10^. The drug revealed numerous merits over conventional antidepressants, including fewer sexual adverse effects than VFX, beneficial effects on sleep disruptions in depression, and independence from side effects like weight gain and serotonin syndrome^11^.

Thus, there seems to be a clinical justification for combining these antidepressants to manage the various symptoms of depressive syndrome. AGM is a favored medication in combination therapy with SSRIs or SNRIs due to its high tolerance and safety profile in comparison to other antidepressants^12^. Furthermore, although VFX and AGM have different mechanisms of action, they seem to work best together when combined^13^. Additionally, a proportion of patients who respond insufficiently to VFX or AGM alone may benefit from their use in combination due to their relative safety and lower risk of drug interactions^14,15^. To sum up, the combination of AGM and VFX for major depression was found to have a good therapeutic rationale, better tolerability, and fewer side effects^14^.

A trial-and-error dose titration strategy is frequently used in clinical practice to help determine an individual’s optimal dose for antidepressant medications. However, therapeutic drug monitoring^16–18^ is a broadly utilized method for determining an individual patient’s ideal dosage, which always improves efficacy and safety when utilized with antidepressant medications.

To date, few reports were applied to analyze AGM in its pharmaceuticals and in biological fluids. These reports included spectrofluorimetry^19,20^, HPLC–UV^21^, and LC–MS^22^. Moreover, various analytical methods were developed for the estimation of VFX in different matrices^23–25^. However, up to our knowledge, no reports were found for the concurrent determination of the co-administered drugs, AGM and VFX, either in their pharmaceuticals or biological fluids. This calls for the development of an appropriate approach for their simultaneous determination within their therapeutic drug levels.

The spectrofluorimetric approach is well-known for its outstanding sensitivity and selectivity^26,27^, although selectivity issues can emerge when determining multi-component samples due to the broadband spectrum overlaps. Synchronous fluorescence spectroscopy (SFS) plays a crucial role when analyzing mixtures with overlapped spectra^28,29^. SFS has been shown to be extremely efficient at characterizing and quantifying a huge variety of compounds^30^. The improved spectral resolution, better selectivity, reduced light scattering, and simplicity of SFS spectra make it preferable to the traditional fluorescence spectroscopy^31^. SFS has been applied for the successful quantitative estimation of various compounds in just one run, thanks to its narrow and sharp spectra^32–34^.

Consequently, our aim was to introduce a smart, sensitive, and environmentally friendly method for the simultaneous quantitation of VFX and AGM in their pharmaceuticals and biological fluids. The need to apply a sustainable method that was scientifically justified and eliminated the consumption of hazardous substances and solvents while still having a lower environmental impact was also taken into consideration. With the least degree of environmental harm, we were able to determine VFX and AGM using the well-established SFS method. A considerable micellar enhancement of the fluorescence intensities of the studied drugs using sodium dodecyl sulfate (SDS) as a micellar system was also achieved. The method could be applied for the concurrent estimation of the above-mentioned drugs in both pharmaceuticals and biological fluids at parts per billion levels. Furthermore, the strategy is simple, hazardous solvent-free, and affordable since it employs a technique that is available in the majority of research laboratories.

## Experimental

### Chemicals and reagents

VFX (98.0%) was purchased from Wako Pure Chemicals Ltd. (Osaka, Japan), while AGM (98.0%) was purchased from Tokyo Chemical Industries Co. (Tokyo, Japan). Britton-Robinson buffer, Brij-35, cetrimide, β-cyclodextrin, sodium dodecyl sulfate, tween-80, ethanol, methanol, and acetonitrile were obtained from Sigma-Aldrich (St. Louis, MO, USA).

Efexor XR® capsules (75 mg VFX/capsule, Batch #FJ4605, Pfizer Ireland Pharmaceuticals, Newbridge Co. Kildare, Republic of Ireland), and Agovald® tablets (25 mg AGM/tablet, Batch #M1120121-8–24, Mash Premiere Co., New York, USA), were purchased from an Egyptian local Pharmacy. For human plasma samples, they were provided by Mansoura University Hospitals (Mansoura, Egypt) and kept at − 20 °C until use before being gently thawed. Britton-Robinson buffer (0.02 M) was prepared in double distilled water to cover pH ranges from 2 to 12. Additionally, organized media solutions at a 1.0% *v/v* or *w/v* concentration were prepared.

### Instrumentation

The measurements of synchronous fluorescence spectra were carried out using an Agilent Cary Eclipse Fluorescence Spectrometer (Agilent Technologies, Santa Clara, CA 95,051, United States). The apparatus was run at a high voltage setting of 800 V with a slit width of 5 nm. At Δλ = 20 nm, spectra were recorded using a scan range of 200–400 nm. Other equipments utilized in the study included an ultrasonic bath (SS 101H 230, USA), a centrifuge (2-16P, Germany), a vortex mixer (IVM-300p, Gemmy Industrial Corp., Taiwan), and a pH-meter (NV P-901, Belgium). Membrane filters (0.45 mm) were obtained from Phenomenex, USA.

### Preparation of standard solutions

Stock solutions of AGM and VFX were prepared separately, at a concentration of 100.0 μg/mL, by dissolving 10.0 mg of each medication in 100.0 mL of double-distilled water as a final volume. After that, working solutions were obtained by further diluting the stock solutions with water. The resulting solutions could be stable for at least a week when kept at 4 °C.

### Construction of calibration graphs

Aliquots of stock solutions were transferred into a set of 5-mL volumetric flasks, with concentration ranges of 20.0–1000.0 and 5.0–200.0 ng/mL for VFX and AGM, respectively. One milliliter of the 1% SDS solution was then added to the solutions, which were then properly diluted with double-distilled water and carefully mixed. Both medications were scanned at Δλ = 20 nm with 5 nm windows over the 200–400 nm range. The synchronous fluorescence intensities (SFIs) were measured at 276 nm and 328 nm for VFX and AGM, respectively. Similarly, blank samples were created. The corrected SFI against the drug's final concentration (ng/mL) was plotted to get the calibration graphs and develop the regression equations.

### Analysis of AGM/VFX synthetic mixture

Aliquots of each drug's stock solution were transferred to 5 mL flasks to create five synthetic mixtures with different ratios and within the linear range of VFX and AGM. After adding one milliliter of 1% SDS, the volume was adjusted using double-distilled water. The measurements were performed as described in section “Construction of calibration graphs”. The concentrations were determined using the respective regression models.

### Analysis of AGM/VFX in commercial dosage forms

The equivalent of ten Agovald® tablets or Efexor XR® capsules’ content were individually weighed and finely ground. Separately, an exact amount of the powder equal to 10.0 mg of AGM or VFX was put into small flasks, and then 50 mL of double distilled water was added. The solutions were sonicated for 20 min, then filtered into clean dry 100-mL volumetric flasks and filled to the desired volume with the same solvent. The above-mentioned procedure was carried out after transferring progressively increasing amounts of the filtrate into 5-mL volumetric flasks. The corresponding regression equations were used to determine the nominal contents of tablets or capsules.

### Construction of calibration curves for the studied drugs in spiked human plasma

Separately, 1.0 mL of human plasma was put into a set of 15 mL of centrifuge tubes. Plasma samples were spiked with aliquots from the VFX and AGM solutions, separately, to achieve final concentrations in the range of 50.0–200.0 and 5.0–15.0 ng/mL, respectively. The solutions were then vortexed for 2 min, followed by protein precipitation by completing up to 5 mL with methanol. After that, the tubes were centrifuged at 6000 rpm for 15 min to allow the medicines to phase separate from the plasma’s contents. One milliliter aliquots of the filtered supernatant were then transferred to 5 mL volumetric flasks. Subsequently, 1 mL of 1% SDS was added, followed by distilled water to complete it to the mark.

Quality control samples should be obtained from different stock solutions by spiking human plasma with a known quantity within the range of drug linearity. As previously mentioned, the corrected SFIs were measured in parallel with a blank sample. The corrected SFIs were then plotted against the concentrations of each drug to generate regression equations.

All experiments were performed in accordance with the Institutional Ethics Approval of the relevant University Committee (Committee of Research Ethics in the Faculty of Pharmacy, Kafrelsheikh University, Kafrelsheikh, Egypt).

### Analysis of AGM/VFX in spiked human plasma

The previously described procedure was repeated in five replicates on a part of the produced supernatant. The corresponding regression equation was used to determine the nominal concentration of the tested drug mixtures in the spiked plasma sample. Simultaneously, a blank experiment was performed on the plasma sample without the addition of the drug solution.

### Ethics declarations

All experiments were performed in accordance with relevant guidelines and regulations and this work was approved by the Committee of Research Ethics in the Faculty of Pharmacy, Kafrelsheikh University, Kafrelsheikh, Egypt (protocol code KFS-Ph-001-22).

### Informed consent

A waiver for the informed consent for the current study was obtained from the Committee of Research Ethics in the Faculty of Pharmacy, Kafrelsheikh University, Kafrelsheikh, Egypt.

## Results and discussion

### Spectral characteristics

**Figure 2:**
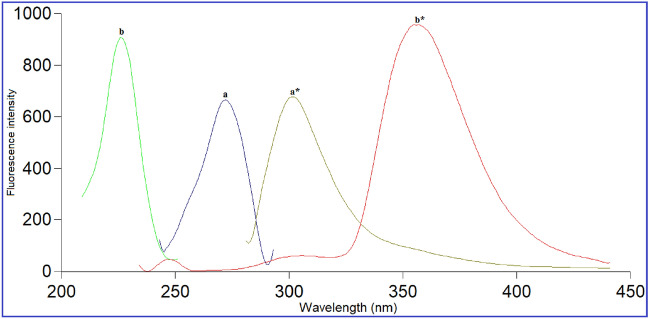
Excitation and emission spectra of VFX (1000.0 ng/mL) (a, a*), and AGM (4.0 ng/mL) (b, b*).

VFX and AGM aqueous solutions exhibit native fluorescence at λ_em_ of 301 and 357 nm following excitation at 272 and 226 nm, respectively. Consequently, the overlap between their emission fluorescence spectra precludes their simultaneous estimation (Fig. 2). The synchronous fluorescence spectroscopy (SFS) was so far the suitable option for analyzing the two drugs simultaneously in just one scan. SFS offers additional merits including enhanced selectivity, spectral overlap reduction, simplification, and its sharp and narrow spectrum with a considerable tolerance for exogenous contaminants, especially when analyzing biological matrices and dose forms. Accordingly, the SFS method was optimized to reduce the spectral overlap and increase the sensitivity.

### Optimization of the experimental conditions

By using the one-factor-at-a-time (OFAT) strategy (changing one single factor at a time while keeping the others constant), several experimental factors that influence the SFI of the concerned drugs (including Δλ change**,** diluting solvents, pH, and organized media) were evaluated and optimized.

#### Effect of Δλ

**Figure 3:**
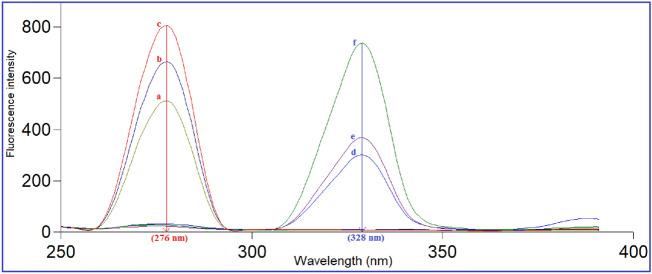
Synchronous fluorescence spectra of (1) a-c, VFX (600.0, 800.0, and 1000.0 ng/mL) (2) d-f, AGM (80.0, 100.0, and 200.0 ng/mL) at Δλ = 20 nm.

Changing Δλ has an obvious impact on peak resolution and sensitivity. The signal strength, spectral structure, and width of the synchronous band are all directly affected. On a large Δλ scale (10–140 nm), the SFS of VFX and AGM were explored. The SF spectra of the two drugs were acquired in a single run with sufficient sensitivity using Δλ of 20 nm, which was shown to be appropriate and give reliable results for both drugs. The well-resolved spectra generated at 276 nm for VFX and 328 nm for AGM allowed for the separation of the two drugs without interference when they were simultaneously analyzed. Therefore, the optimal wavelength for both drugs was found to be Δλ = 20 nm (Fig. 3). Lower Δλ than this value revealed reduced SFI for both VFX and AGM, while values above 20 nm resulted in overlapping spectra. Consequently, Δλ of 20 nm was further used in the investigation.

#### Effect of diluting solvents

**Figure 4:**
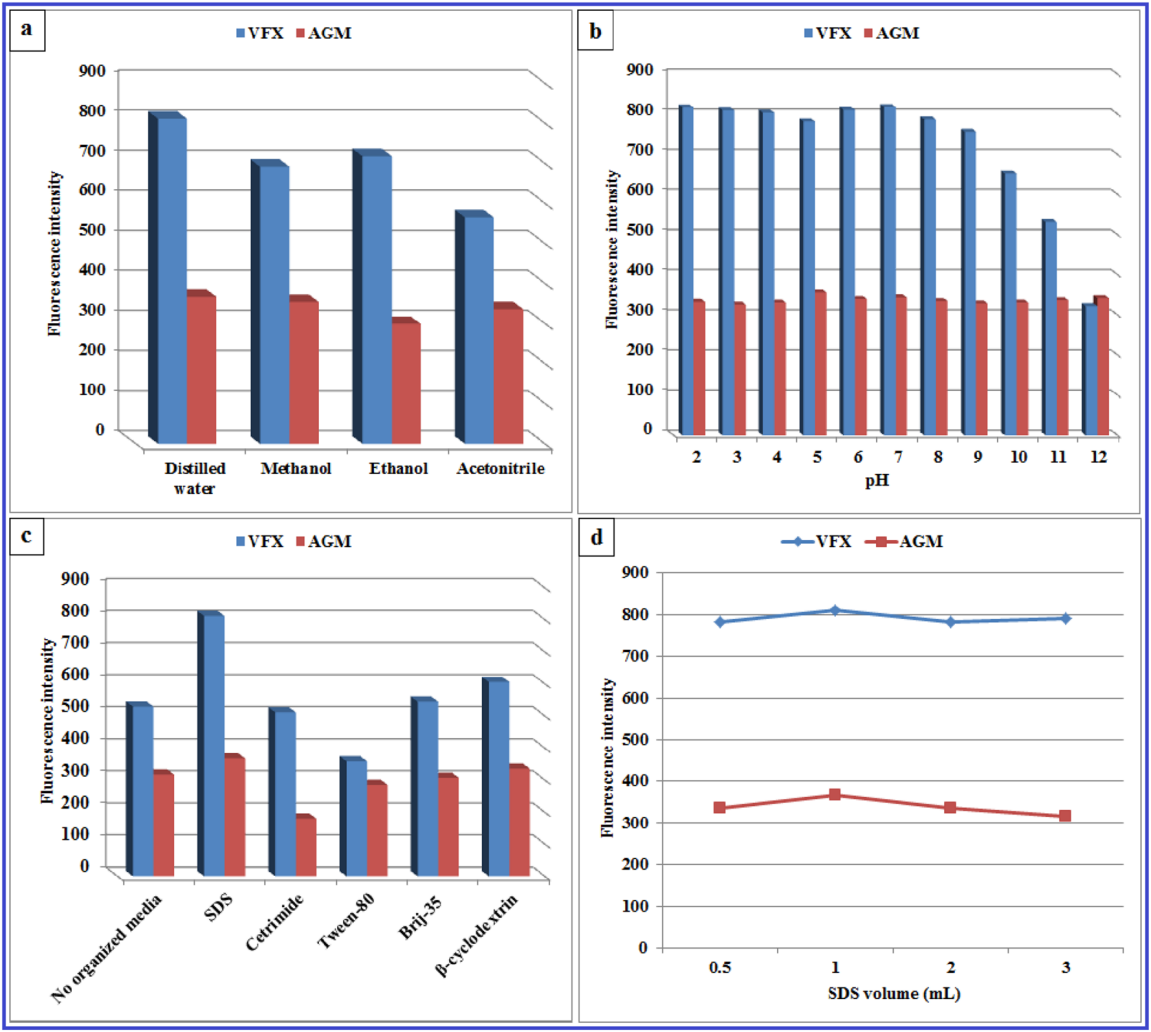
Influence of different diluting solvents (**a**), pH (**b**), organized media (**c**), and SDS volume (**d**) on the SFI of 1000.0 ng/mL VFX and 100.0 ng/mL AGM.

Various diluting solvents such as double distilled water, acetonitrile, methanol, and ethanol were investigated. The greatest SFI for both VFX and AGM with high resolution was obtained by water (Fig. 4a), which gives the proposed method an additional benefit in terms of sustainability. This impact rises with increasing solvent polarity. In contrast to nonpolar molecules, only polar fluorophores often show a stronger affinity to solvent polarity^27^. The dielectric constant (Ɛ) is a measure of a solvent's polarity, the greater the dielectric constant, the more polar the solvent^35^. Moreover, AGM and VFX are polar enough to exhibit high SFI in double distilled water. On the other hand, acetonitrile, methanol, and ethanol produced high blank readings and reduced the SFIs of the two drugs (Fig. 4a). Therefore, water was used as the diluting solvent of choice.

#### Effect of pH

The SFI of both AGM and VFX aqueous solutions over a wide pH range (2–12) was investigated using 0.02 M Britton-Robinson buffer. No significant effect was observed upon changing the pH, either acidic or alkaline. Moreover, the SFI of VFX gradually decreased upon increasing the pH above 7 (Fig. 4b). Therefore, no buffer was used in the consequences.

#### Effect of organized media

The ability of various organized media to improve the SFI of AGM and VFX was evaluated at a concentration higher than their critical micelle concentrations (CMC)^34^. They included SDS, cetrimide, tween-80, Brij-35, and β-cyclodextrin. SDS was found to significantly enhance the SFIs of VFX and AGM by about 53% and 16% of their native fluorescence, respectively (Fig. S1, Supplementary Material). The enhancement in this micelle is obviously caused by the inhibition of a nonradiative process, which can be attributed to one or more of the following: sequestering from water, resulting in a considerable decrease in the polarity of the fluorophore’s microenvironment as well as an increase in the micro-viscosity. This explains why the addition of 1% *w/v* SDS significantly improved the SFI of AGM and VFX. On the other hand, cetrimide was found to significantly decrease the FI of both drugs (Fig. 4c). As for Brij-35 and β-cyclodextrin, no enhancement was observed, and interfering peaks were detected at 285 and 278 nm, respectively. For tween-80, even an enhancement of the SFI of AGM was observed; a high blank reading was measured with interfering peaks at 321 and 282 nm. As a result, SDS was used in the consequences for the selective enhancement of the SFI of both drugs. Consequently, different volumes of SDS were investigated over the range of 0.5–3.0 mL. One milliliter of 1% SDS was found the optimal for the highest SFI of both drugs (Fig. 4d).

### Method validation

The proposed method was validated according to the ICH guidelines^36^. Validation studies included linearity and range, precision, accuracy, limits of detection (LOD) and quantitation (LOQ), and selectivity.

Firstly, the method exhibited excellent linearity (*r*^*2*^ > 0.999, for both drugs) over the ranges of 20.0–1000.0 and 5.0–200.0 ng/mL for VFX and AGM, respectively (Fig. S2, supplementary material). Moreover, both LOD and LOQ were calculated from 3.3 × (*S*_*a*_/b), and 10 × (*S*_*a*_/b), respectively, where *S*_*a*_ is the standard deviation of the intercept and b is the slope of the calibration curves. The obtained low LODs and LOQs (Table 1) confirm the high sensitivity of the proposed method (at parts per billion levels). Therefore, the method was efficiently applied for the simultaneous analysis of the two co-administered drugs in human plasma.

**Table 1:** Analytical performance data for the proposed method.

Parameters	VFX	AGM
Wavelength (nm) at Δλ = 20 nm	276	328
Linearity range (ng/mL)	20.0–1000.0	5.0–200.0
Intercept (a)	40.71	10.23
Slope (b)	0.7727	3.624
Correlation coefficient (*r*^*2*^)	0.9998	0.9999
S.D. of the residuals (*S*_*y/x*_)	1.181	1.0006
S.D. of the intercept (*S*_*a*_)	0.197	0.157
S.D. of the slope (*S*_*b*_)	0.0012	0.0059
% RSD	0.557	1.165
% Error	0.19	0.41
LOD (ng/mL)	0.84	0.14
LOQ (ng/mL)	2.55	0.43

**Figure 5:**
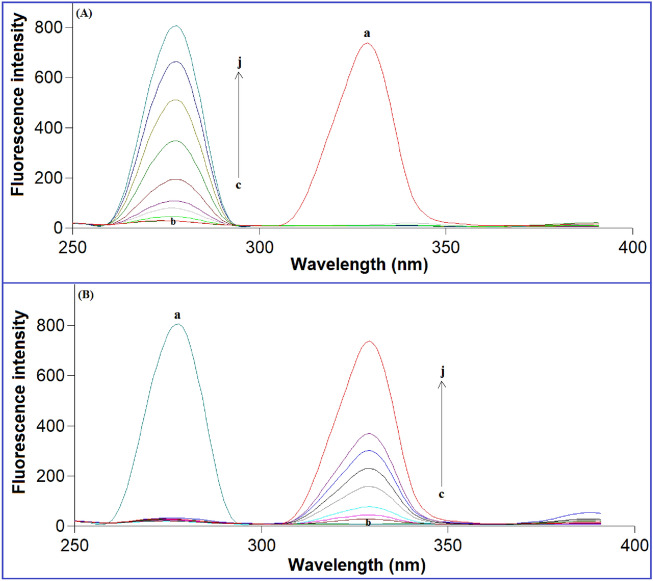
Synchronous fluorescence spectra of: (**A**) (a) AGM (200.0 ng/mL), (b) Blank, and (c-j: 20.0, 50.0, 100.0, 200.0, 400.0, 600.0, 800.0, 1000.0 ng/mL VFX) at Δλ = 20 nm, (**B**) (a) VFX (1000.0 ng/mL), (b) Blank, and (c-j: 5.0, 10.0, 20.0, 40.0, 60.0, 80.0, 100.0, 200.0 ng/mL AGM) at Δλ = 20 nm.

The method’s precision and accuracy were also evaluated as intra- and inter-day precisions through the analysis of three different concentrations within the same day or on three consecutive days, respectively. Based on the obtained results, the % RSD was calculated to express the precision, while the mean %recovery of each nominal concentration (three replicates) demonstrated the method's accuracy. The low %RSD revealed the excellent precision of the proposed method (Table 2), and the great recovery demonstrated its high accuracy (Table 3). Additionally, the selectivity of our method was also evaluated through the simultaneous estimation of the studied drugs at the same time without any interference from each other (Fig. 5). The ability of the proposed method to analyze both drugs in spiked human plasma with low %RSD and excellent recovery and without any interference from the plasma components demonstrated the high selectivity of the proposed method.

**Table 2:** Precision data for the determination of VFX and AGM by the proposed method.

	Conc. (ng/mL)	Inter-day precision		Intra-day precision	
		% Recovery ± S.D.	%RSD	% Recovery ± S.D.	% RSD
VFX	100.0	99.55 ± 0.73	0.733	97.95 ± 0.84	0.857
	400.0	100.14 ± 0.46	0.459	99.47 ± 0.92	0.924
	800.0	99.68 ± 0.87	0.872	100.62 ± 1.23	1.222
AGM	10.0	100.23 ± 0.43	0.429	100.46 ± 0.77	0.766
	40.0	99.29 ± 0.75	0.755	99.44 ± 0.85	0.855
	80.0	99.89 ± 0.48	0.480	98.85 ± 0.81	0.819

N.B. Each reading is the average of three separate determinations.

**Table 3:** Accuracy results of the proposed method for the determination of VFX and AGM in pure forms.

Parameters	VFX			AGM		
	Amount taken (ng/mL)	Amount found (ng/mL)	% Recovery	Amount taken (ng/mL)	Amount found (ng/mL)	% Recovery
	20.0	19.79	98.96	5.0	4.90	98.02
	50.0	49.56	99.12	10.0	9.94	99.43
	100.0	100.03	100.03	20.0	19.94	99.68
	200.0	197.09	98.55	40.0	40.21	100.53
	400.0	397.69	99.42	60.0	60.63	101.05
	600.0	607.34	101.22	80.0	80.19	100.23
	800.0	806.64	100.83	100.0	98.98	98.98
	1000.0	991.71	99.17	200.0	200.22	100.11
Mean (${\overline{\text{X}}}$)			99.22			99.74
± S.D.			0.55			1.16
% RSD			0.557			1.165

N.B. Mean of three separate determinations.

### Application to VFX and AGM synthetic mixtures

The proposed method was utilized to evaluate synthetic mixtures with varying VFX and AGM ratios (Fig. S3, supplementary material). The concentrations of both drugs in various ratios were estimated using the respective regression equations. The findings produced demonstrated the method’s accuracy, as shown in Table S1, supplementary material.

### Application to pharmaceutical dosage forms

The optimized method was applied for the estimation of VFX and AGM in their single dosage forms. VFX was analyzed in its 75 mg capsules, while AGM was determined in its 25 mg tablets. The method exhibited high % recoveries (> 98%) with low %RSD (< 1.62) (Table 4), confirming its excellent applicability for the determination of VFX and AGM in their pharmaceuticals without interference from the co-formulated excipients.

**Table 4:** Determination of VFX and AGM in pharmaceutical dosage forms using the proposed method.

Dosage forms	Amount taken (ng/mL)	Amount found (ng/mL)	% Recovery
Efexor XR® capsules (75 mg VFX/capsule)	100.0	98.96	98.96
	200.0	203.39	101.70
	400.0	401.83	100.46
	800.0	785.64	98.20
	1000.0	1010.18	101.02
Mean			99.83
SD			1.56
% RSD			1.564
Agovald® tablets (25 mg AGM/tablet)	10.0	9.79	97.94
	20.0	19.73	98.67
	40.0	40.47	101.18
	80.0	80.57	100.72
	100.0	99.43	99.43
Mean			99.63
SD			1.57
% RSD			1.573

N.B. Each result is the average of three separate determinations.

### Application to spiked human plasma

The optimized method was used to simultaneously analyze VFX and AGM in human plasma. AGM has a high bioavailability of more than 78% and is easily absorbed after oral administration^10^. Its maximum plasma concentration (C_max_) is reported to be 15.1 ng/mL^37^. Moreover, the therapeutic plasma level of VFX was reported in the range of 30–200 ng/mL^38^. Given that these concentrations fall within the established method's linear range, it is concluded that this method can concurrently determine both drugs in human plasma. By graphing the SFI *versus* the drug concentration in ng/mL, a linear relationship was generated in plasma samples spiked with VFX and AGM using the optimized method. The following equations were generated through a linear regression analysis of the data:

$${\text{SFI}}\, = \,{1}.{\text{6114C}} - {3}0.{5}0{9 }\left( {r^{2} \, = \,0.{9993}} \right){\text{ for VFX,}}$$

$${\text{SFI}}\, = \,{4}.{\text{5517C}}\, + \,{1}.{4828 }\left( {r^{2} \, = \,0.{9981}} \right){\text{ for AGM}}.$$

A linear range was obtained by spiking plasma samples with VFX and AGM at different concentrations within the ranges of 50.0 − 200.0 and 5.0 − 15.0 ng/mL, respectively. The obtained high % recoveries (> 97%) demonstrated the high efficiency of our method in such complex matrices (Table 5).

**Table 5:** Application of the proposed method for the determination of the studied drugs in spiked human plasma samples.

Parameter	VFX			AGM		
	Amount taken (ng/mL)	Amount found (ng/mL)	% Recovery	Amount taken (ng/mL)	Amount found (ng/mL)	% Recovery
	50.0	48.721	97.44	5.0	4.947	98.94
	75.0	76.647	102.20	8.0	8.023	100.29
	100.0	98.988	98.99	10.0	10.219	102.20
	150.0	151.737	101.16	12.0	11.758	97.98
	200.0	198.901	99.45	15.0	15.053	100.35
Mean			99.95			99.85
SD			2.14			1.83
% RSD			2.142			1.832

N.B. Each result is the average of three separate determinations.

### Assessment of method greenness by various metrics tools

The development of a variety of tools and related metrics to assess the environmental effects of various analytical procedures has preceded the rise in efforts to develop greener analytical techniques^39^. Although each published tool has its own computation and criteria, these tools and metrics might point chemists in the direction of more environmentally friendly methods^40^. Therefore, comparing both on an equal footing might help scientists decide whether to employ one over the other, depending on their goals and the level of precision they are looking for in these instruments^39^. In this context, we aim to evaluate the greenness of our proposed method using different metrics, including the Green Analytical Procedure Index (GAPI)^41^ and Analytical GREEnness (AGREE)^42^.

**Figure 6:**
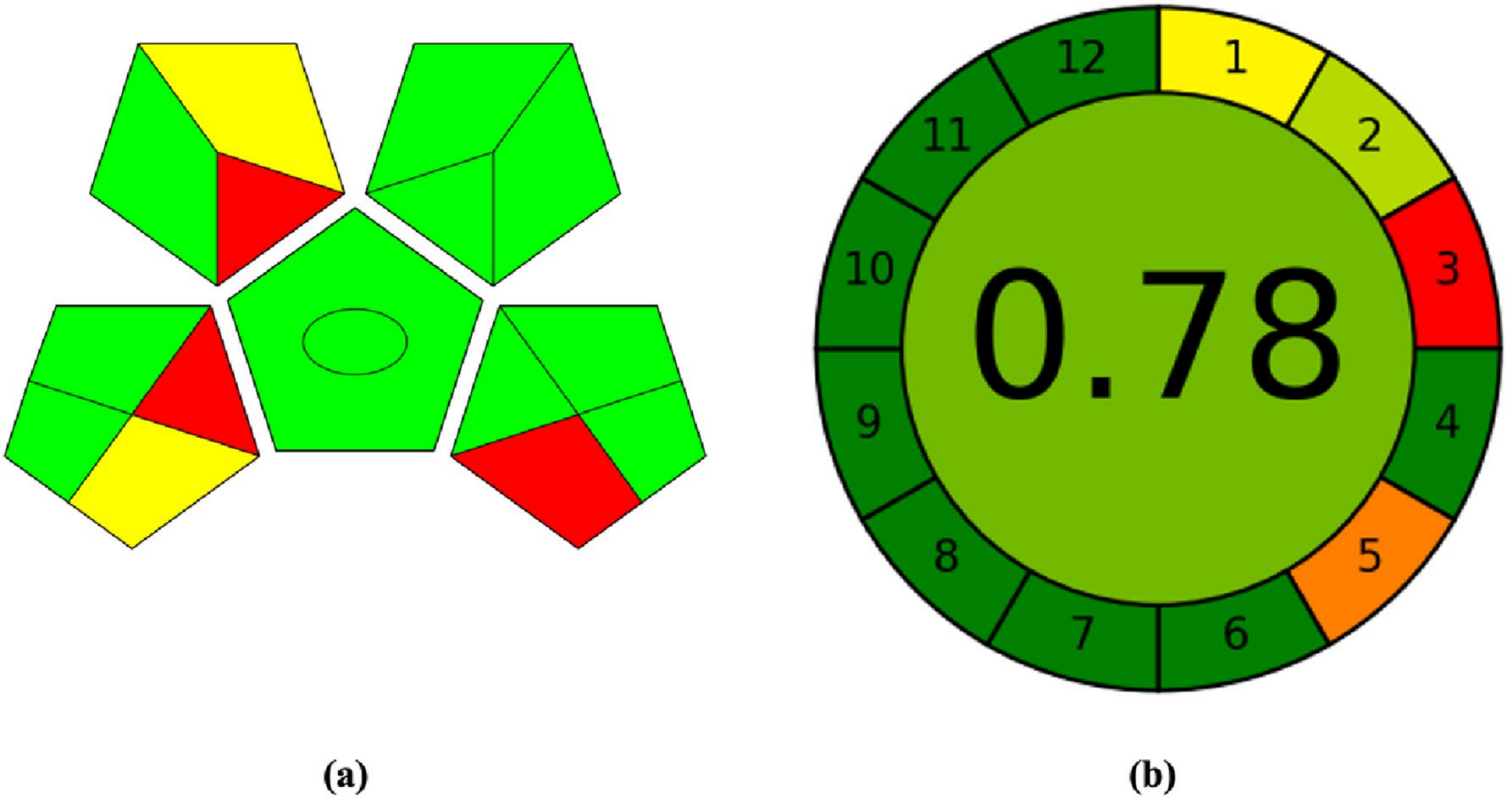
Assessment of the greenness profile of the proposed method by (**a**) GAPI, (**b**) AGREE.

The GAPI was recently built for the evaluation of the whole analytical method. It is made up of five pentagrams that represent sample collection and preparation, chemicals and solvents, instruments, and technique type. The use of red color indicates that this step may be environmentally hazardous, yellow color indicates that this step has a moderate environmental effect, and green color indicates that this step is environmentally benign. From the step sample collection until the final determination, GAPI was utilized to demonstrate the proposed method’s green potential. The GAPI pictogram for the proposed procedure is presented in Fig. 6a. The only drawback is the pretreatment step in the case of plasma which uses a small amount of methanol for protein precipitation. Additionally, AGREE, a metric tool that emphasizes sample preparation, was used to assess the new method's greenness. It is based on 12 impact categories, which were then converted to subs-cores on a 0–1 scale and used to produce the assessment's final result. The factors for evaluation included sample size, throughput, waste output, energy consumption, and the choice and use of solvents, materials, and reagents. The ability to differentiate between the relevance of criteria by giving weights to them was also used in the evaluation. The findings of the AGREE analysis are presented in Fig. 6b. On a scale of 0 to 1, the AGREE analysis of the studied compounds received a general score of 0.78, confirming the excellent greenness and lower environmental impact of our procedure.

## Conclusion

The first synchronous fluorescence spectroscopic method was developed for the simultaneous estimation of venlafaxine and agomelatine. The method relies on the use of water rather than any toxic or hazardous solvents. Additionally, the addition of an SDS micellar system greatly improved the method's sensitivity. The method was fully validated and exhibited low LODs in parts per billion levels. Consequently, the method could be employed for therapeutic drug monitoring in spiked human plasma. The greenness of the method was evaluated using various metric tools, confirming its excellent greenness and low environmental impact. The method has the pros of being simple, easy, green, precise, and highly sensitive. The proposed method's advantages have all demonstrated its effectiveness and suitability for use in pharmaceutical quality control laboratories.

## Supplementary Information

Supplementary Information.

## Data Availability

The datasets generated and/or analyzed during the current study are available from the corresponding author upon reasonable request.
