# Supplementary material for: Green synchronous spectrofluorimetric method for the simultaneous determination of agomelatine and venlafaxine in human plasma at part per billion levels
Source: Sci Rep. 2022 Dec 29;12:22559. doi: 10.1038/s41598-022-26827-2 (PMC9800581; doi:10.1038/s41598-022-26827-2)
Supplement: Supplementary file 1 — Supplementary Information. [file 41598_2022_26827_MOESM1_ESM.pdf]

Green Synchronous Spectrofluorimetric Method for the Simultaneous Determination of Agomelatine and Venlafaxine in Human Plasma at Part per Billion Levels

Galal Magdy [1*], Fathalla Belal [2], Asmaa Kamal El-Deen [2*]

[1] Pharmaceutical Analytical Chemistry Department, Faculty of Pharmacy, Kafrelsheikh University, Kafrelsheikh, 33511, Egypt

[2] Pharmaceutical Analytical Chemistry Department, Faculty of Pharmacy, Mansoura University, Mansoura, 35516, Egypt

[*]Corresponding authors:

Asmaa Kamal El-Deen, E-mail: asmaakamal91@mans.edu.eg, Tel.: +201552215860

Galal Magdy, E-mail: galal_magdy@pharm.kfs.edu.eg, Tel.: +201000137394

Supplementary Figures Captions

Fig. S1 Synchronous fluorescence spectra of VFX (1000.0 ng/mL) and AGM (200.0 ng/mL), where: (a, b) without SDS, and (a*, b*) with 1 mL of 1.0 % *w/v* SDS

Fig. S2 Calibration graphs for the synchronous spectrofluorimetric determination of VFX (a) and AGM (b) by the proposed method

Fig. S3 Synchronous fluorescence spectra of (a). VFX (400.0 ng/mL), (b). AGM (200.0 ng/mL), (c). a synthetic mixture of VFX/AGM at $\Delta \lambda = 20$ nm

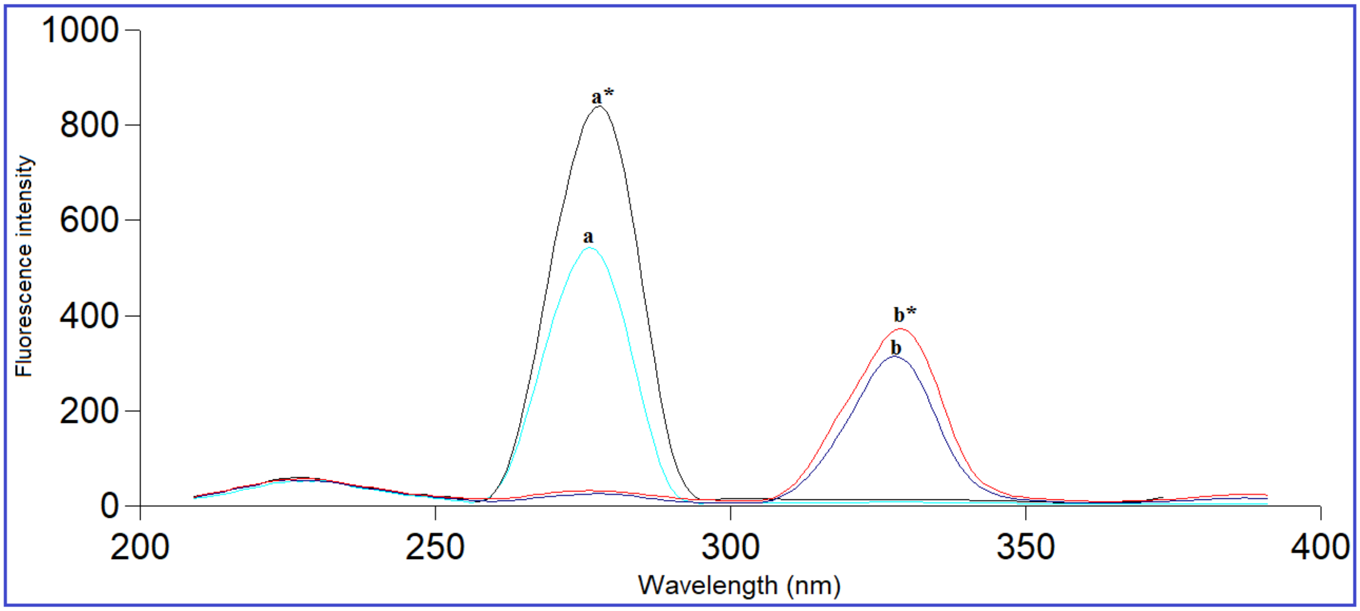

1000
800
600
400
200
0
Fluorescence intensity
a*
a
b*
b
200
250
300
350
400
Wavelength (nm)

Fig. S1

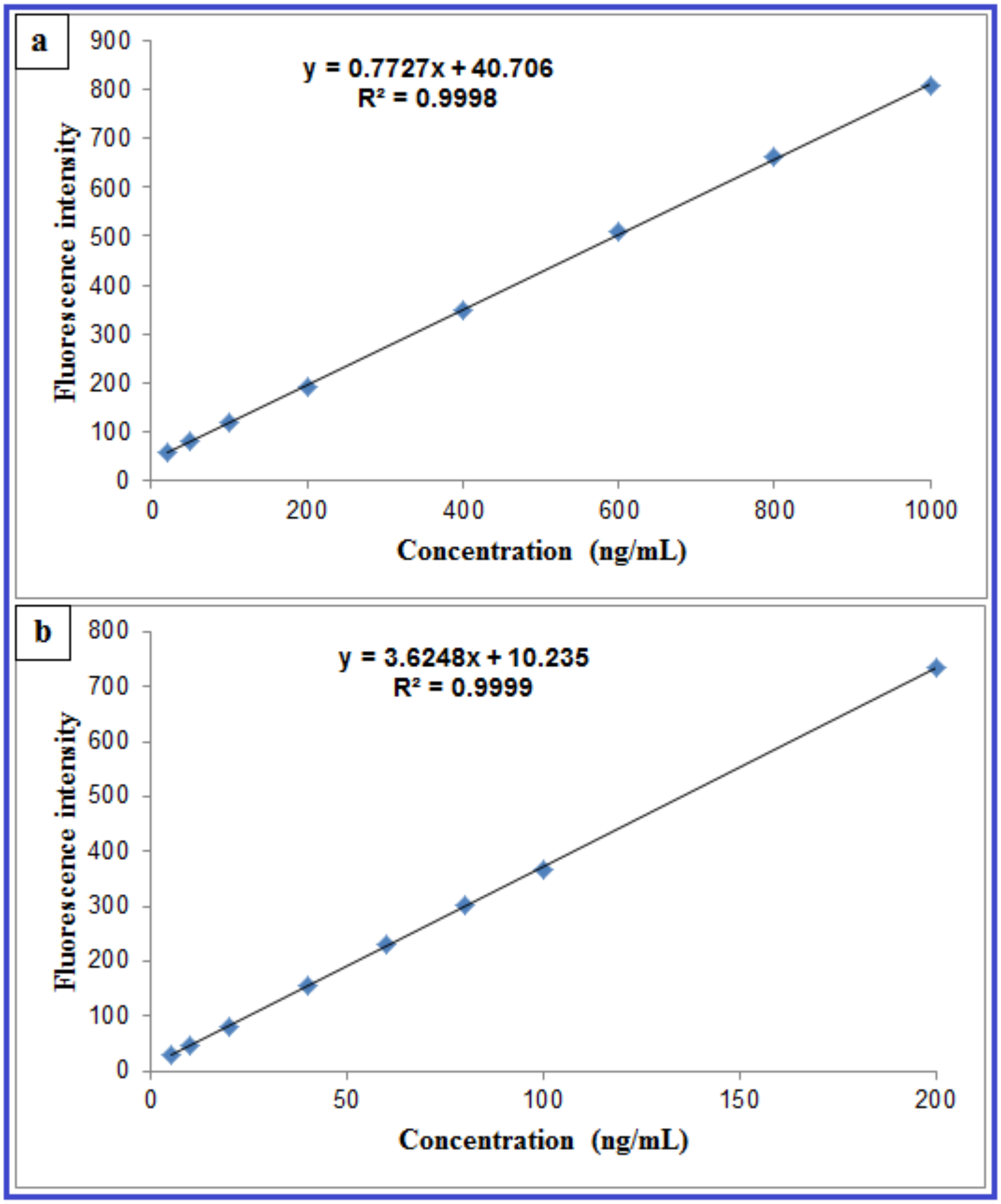
a
y = 0.7727x + 40.706
R² = 0.9998
Fluorescence intensity
Concentration (ng/mL)
0
100
200
300
400
500
600
700
800
900
0
200
400
600
800
1000
b
y = 3.6248x + 10.235
R² = 0.9999
Fluorescence intensity
Concentration (ng/mL)
0
100
200
300
400
500
600
700
800
0
50
100
150
200

Fig. S2

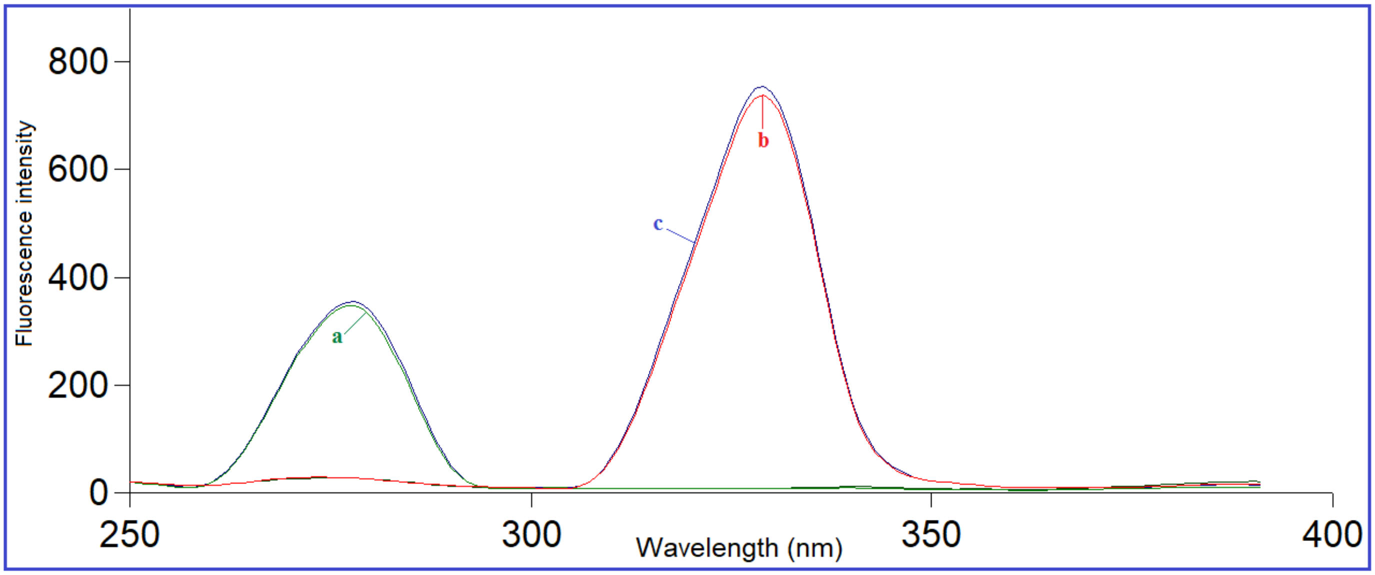

800
600
400
200
0
Fluorescence intensity
250
300
350
400
Wavelength (nm)
a
b
c

Fig. S3

Supplementary Table

Table S1: Assay results for the determination of VFX and AGM in synthetic mixtures using the proposed method

Mix. Number	Amount taken (µg/mL)		Amount found (µg/mL)		%Recovery	
	VFX	AGM	VFX	AGM	VFX	AGM
**1**	0.05	0.1	0.049	0.099	98.60	99.30
**2**	0.4	0.2	0.402	0.202	100.63	101.20
**3**	0.2	0.2	0.199	0.199	99.65	99.70
**4**	0.8	0.08	0.799	0.080	99.88	100.75
**5**	0.02	0.2	0.020	0.198	99.50	99.15
**Mean**					99.60	99.73
**± SD**					0.83	0.72
**% RSD**					0.834	0.724

N.B. Each reading is the average of three separate determinations.
